# Myelin Damage in Diffuse Axonal Injury

**DOI:** 10.3389/fnins.2019.00217

**Published:** 2019-03-19

**Authors:** Jiao Mu, Meiyu Li, Tingting Wang, Xiujuan Li, Meiling Bai, Guohui Zhang, Jiming Kong

**Affiliations:** ^1^Department of Forensic Medicine, Hebei North University, Zhangjiakou, China; ^2^Department of Human Anatomy and Cell Science, College of Medicine, University of Manitoba, Winnipeg, MB, Canada

**Keywords:** diffuse axonal injury, demyelination, oligodendrocyte, axon-myelin unite, corpus callosum, brain stem

## Abstract

Diffuse axonal injury (DAI) is characterized by delayed axonal disconnection. Although the effect of DAI on axonal pathology has been well documented, there is limited information regarding the role of myelin in the pathogenesis of DAI. We used a modified Marmarou method to create a moderate DAI model in adult rat and examined the corpus callosum and brain stem for myelin pathology and dynamic glial responses to DAI. During the first week following DAI, Luxol Fast Blue staining and western blot analysis for MBP showed significant loss of myelin in the corpus callosum and the brain stem. Increased apoptosis of mature oligodendrocyte, as depicted by its marker CC-1, was observed. Conversely, there was an increased number of Olig2-positive cells accompanied by hypertrophic microglia/macrophage and mild reactive astrocytes. Electron microscopy revealed degenerating axons in the corpus callosum and marked myelin abnormalities in the brain stem in the early stage of DAI. Brain stem regions exhibited myelin intrusions or external protrusions with widespread delamination and myelin collapse, leading to degeneration of accompanying axons. Our results show distinct pathologic processes involving axon and myelin between the corpus callosum and the brain stem in DAI. Oligodendrocyte selective vulnerability and subsequent demyelination may contribute to axonal degeneration in the brain stem. Defining the cause of ongoing oligodendrocyte death and promoting myelin regeneration may provide important targets for therapeutic interventions of DAI.

## Introduction

Diffuse axonal injury (DAI) is one of the most common and important pathologic features of traumatic brain injury (TBI), which affects millions of people worldwide (Ma et al., 2016). DAI is caused by energy transmitted to the brain via rapid head rotation or deceleration/acceleration, leading to multifocal injury of white matter tracts (Adams et al., 1989; Mu et al., 2015). Development of pathological changes in axons following DAI has been well documented including delayed axotomy caused by both initial mechanical force and complex secondary cascade (Vargas and Barres, 2007; Johnson et al., 2013; Jang and Kwon, 2016). There is, however, limited information regarding the role of myelin and oligodendrocyte in DAI.

In the central nervous system, myelin is a cholesterol-rich extension of oligodendrocyte plasma membrane, which is important for axonal maintenance and function (Aggarwal, 2011). Oligodendrocytes, as the primary cells responsible for generating and maintaining the myelin sheath, are highly vulnerable to various stimuli, including excitotoxicity, oxidative stress, and inflammation (Bradl and Lassmann, 2010). As all the aforementioned stimuli are features of secondary cascade in DAI, it is expected that DAI has an influence on oligodendrocytes and further cause myelin loss consequently.

Indeed, the mixed and intertwined nature of axon and myelin pathology is apparent in DAI. It is understood traditionally that myelin collapse is secondary to axon degeneration (Armstrong et al., 2015). However, in a recent study, Maxwell (Maxwell, 2013) found that in stretch-injury to optic nerve fibers, myelin dislocations occur within 1–2 h after injury and damage to the myelin sheath and oligodendrocytes of the optic nerve fibers may facilitate the continuance of axonal loss. This prompted us to test a hypothesis that myelin damage plays an important role in pathophysiological processes in DAI. Here we report dynamics of demyelination and selective vulnerability of oligodendrocytes in DAI. Our results suggest a complex interplay between axonal damage, oligodendrocyte death and myelin loss in moderate DAI.

## Materials and Methods

### Animals and DAI

All procedures involving animals were approved and monitored by the Animal Care Committee of Hebei North University. Adult male Sprague-Dawley rats (weighting 220–280 g) were included in our study. All rats were housed at five animals per cage on a 12 h light/dark cycle with free access to food and water.

A moderate DAI model was induced by a modified Marmarou method (Zhang et al., 2015). Animals were anesthetized with 5% isoflurane. The scalp of the anesthetized rat was shaved, a midline incision was performed, and the periosteum covering the vertex was exposed. A steel disk of 10 mm in diameter and 3 mm in thickness was fixed at the center of the vertex. Subsequently, rats were prostrated and fixed onto a sponge bed. Injury was delivered by dropping a 450 g weight freely onto the coin from a height of 1 m. Then, the rat was immediately removed, and the scalp was sutured after gently removing the steel disk from the skull. In the control group, rats (*n* = 11) underwent the same surgical procedure without impact. Five survival time-points were established post DAI, with animals euthanized at 1, 2, 3, 5, and 7 days following injury (*n* = 11 in each DAI group).

### Immunohistochemistry, Immunofluorescence, and Histopathology

Once anesthetized, rats (*n* = 3 in each group) were perfused transcardially with 200 mL 0.01 M phosphate buffered saline (PBS) followed by 200 mL 4% paraformaldehyde. Brains were further post-fixed by immersion overnight and then to undertake dehydration, vitrification and embedding. The paraffin-embedded tissues were cut using microtome to provide 5-nm-thick tissue sections for immunohistochemistry, immunofluorescence, and histopathology.

#### Immunohistochemistry

After deparaffinization and rehydration of the brain sections, antigen retrieval was performed with 0.1 M sodium citrate at 100°C for 10 min. Following incubation with 3% hydrogen peroxide for 15 min, the sections were incubated with goat serum to reduce non-specific reaction for 15 min. Then, the sections were incubated with the primary antibody (detailed information in Table 1): olig2 (1:500) or Iba1 (1:2000) at 4°C overnight. The negative control specimens underwent the same procedures, but primary antibodies were replaced by PBS. Following washing with 0.01 M PBS, biotinylated goat anti-rabbit secondary antibody and S-A/HRP reagent were applied subsequently at 37°C for 20 min, respectively. The positive reaction was visualized with DAB, and the section was then dehydrated in graded alcohols, cleared in xylene and covered.

**Table 1:** Overview of primary antibodies.

Antibody	Species	Target	Product number and supplier
MBP	Anti-rabbit	Myelin	ab40390, Abcam, Cambridge, United Kingdom
Olig2	Anti-rabbit	Mature OLs and OPCs	13999-1-AP, Proteintech, Rosemont, IL, United States
CC-1	Anti-mouse	Mature OLs	ab16794, Abcam, Cambridge, United Kingdom
Iba1	Anti-rabbit	Microglia/ macrophages	ab178847, Abcam, Cambridge, United Kingdom
GFAP	Anti-rabbit	Astrocytes	16825-1-AP, Proteintech, Rosemont, IL, United States


*OLs, oligodendrocytes; OPCs, oligodendrocyte precursor cells*.

#### Immunofluorescence

After deparaffinization, rehydration and antigen retrieval, the brain sections were blocked in goat serum for 30 min at 22°C. Then, the sections were incubated with the primary antibody (detailed information in Table 1) – GFAP (1:200) or CC-1 (1:100) at 4°C overnight. The negative control specimens underwent the same procedures, but primary antibodies were replaced by PBS. This was followed by a 2-h incubation at 37°C with secondary antibodies conjugated with either CY3 (1:1000, A0521, Beyotime, Shanghai, China) or FITC (1:1000, bs-0295G-FITC, Bioss, Beijing, China). Sections were then washed three times in PBS and then incubated with DAPI (C1005, Beyotime, Shanghai, China) for 1 min before being coverslipped with a mounting medium.

#### Histopathology

Luxol fast blue (LFB) staining was used to evaluate changes in the structural integrity of myelin. Following deparaffinization and rehydration, slices were placed into an LFB staining solution for 2–4 h at 60°C and then cooled to ambient temperature. Excessive staining was removed by distilled water rinses for 1 min each. The section was dehydrated in graded alcohols, cleared in xylene, and covered.

An Olympus BX-51 fluorescent microscope (Olympus America, Center Valley, PA, United States) connected to a computer by a color CCD camera was used to obtain and edit images. The analysis software (Olympus) was used to acquire images at different magnifications. For GFAP, CC-1, and DAPI examination, the excitation spectra were 495, 550, and 340 nm, respectively, and the emission spectra were 519, 570, and 488 nm, respectively. Cell counting was conducted on nine randomly chosen fields for each sample and measured using Image-Pro Plus software (version 5.1; Media Cybernetics, Inc., Silver Spring, MD, United States).

### CC-1/TUNEL Co-labeling

Cell apoptosis was assessed by TUNEL staining using a cell death detection kit (Roche, Indianapolis, IN, United States). Following deparaffinization and rehydration, the brain sections were processed for TUNEL staining according to the manufacturer’s instruction. For negative control, the sections were incubated with label solution (without terminal transferase) instead of TUNEL reaction mixture. For positive control, the sections were incubated with DNase I recombinant for 10 min at 25°C to induce DNA strand breaks prior to labeling procedures. The same slides were further used for immunofluorescence with a primary antibody to CC-1 ratio of 1:100.

### Western Blot Assessment

Once anesthetized, rats (*n* = 5 in each group) were perfused through the heart with 200 mL saline solution. The homogenates of the corpus callosum (CC) and brain stem were resolved on SDS PAGE and transferred to a nitrocellulose membrane using the Geni blot system (Liuyi Co., China). The membrane was blocked with 5% milk in Tris-buffered saline+tween-20 (TBST) for 2 h at room temperature and then incubated with the Anti-MBP antibody (1:2000); Anti-Olig2 antibody (1:1500); Anti-GFAP antibody (1:7000); and Anti-Iba1 antibody (1:3000) overnight at 4°C. After washing three times for 5 min each with TBST, the membrane was incubated with a corresponding secondary antibody: goat anti-rabbit (A0208, Beyotime, Shanghai, China) and goat anti-mouse (A0216, Beyotime, Shanghai, China), for 60 min at room temperature. After washing, blots were developed with a solution containing 10 ml PBS, 0.025% (v/v) H_2_O_2_, and 8 mg 4-chloro-1-naphthol dissolved in 2 ml of methanol. Specific immunoreactive bands were quantified by computer-assisted densitometry.

### Transmission Electron Microscopy and Quantification

Once anesthetized, rats (*n* = 3 in each group) were perfused through the heart with 200 mL saline solution followed by 250 mL 2% paraformaldehyde and 0.5% glutaraldehyde in 0.01 M PBS. The brainstem and the CC were trimmed into blocks of 2 mm × 1 mm × 0.5 mm and conventionally fixed, rinsed, dehydrated and embedded in epoxy resin. The samples were then cut into 70-nm-thick sections, stained with uranyl acetate and bismuth subnitrate, and examined on a transmission electron microscope (JEOL, Peabody, MA, United States).

Images of brain sections through the CC and brain stem were acquired at 5,000× magnification for measurements of g-ratio. The g-ratio was calculated for each myelinated axon by dividing the average axon diameter by the average total fiber diameter. Additional images were taken at 10,000× for illustration of pathology.

### Statistical Analysis

SPSS 16.0 and Prism 5.0 (GraphPad Prism Software) were used for statistical analyses and graphing of quantitative data. Independent-samples *t*-test analysis of variance was performed to determine significant differences across each post-injury time point compared to the control. Slopes were calculated using linear regression. Image-Pro Plus software was used for cell counting in immunohistochemistry. *P*-values <0.05 were considered statistically significant.

## Results

**FIGURE 1:**
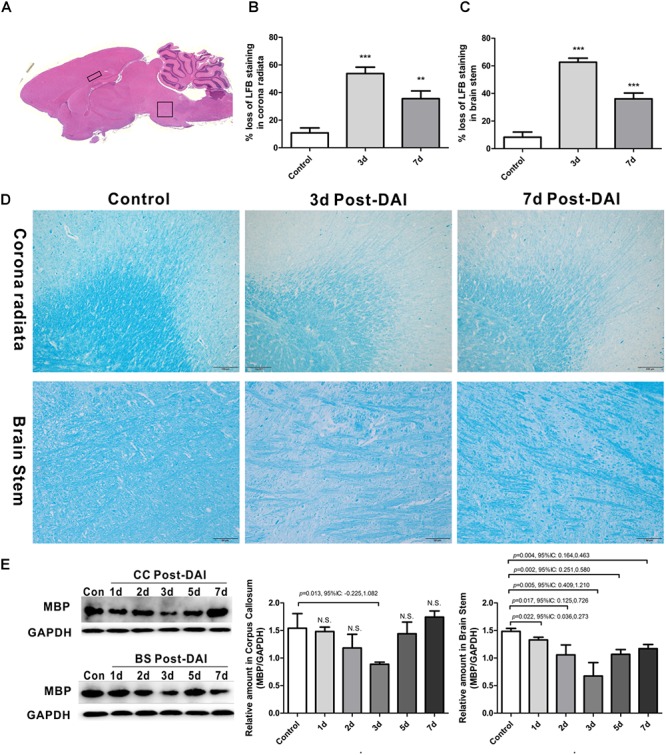
Brain overview and myelin loss in DAI (diffuse axonal injury). **(A)** An overview of brain morphology determined on H&E-stained. Squares show ROIs focused on the CC and brain stem evaluated in the present study; **(B–D)** LFB staining at 3 and 7 days post-injury shows myelin disruption and myelin loss in the corona radiate and brain stem; **(E)** western blot analysis indicates that MBP expression was significantly decreased throughout the first week after injury in brain stem. The CC region exhibits significant decreased MBP expression at 3 days after injury. N.S. indicates no significant changes between DAI group and control group.

The injured rats showed conscious disturbance, decortication flexion deformity of the forelimbs, rigidity of hindlimbs, and momentary respiratory depression or shallow breathing in the DAI group after impact. The mortality was about 20%. No skull fracture, cerebral contusion or focal white matter tears were observed after closed-skull impact in adult rats. Squares show regions of interest (ROIs) focused on the CC and brain stem evaluated in the present study (Figure 1A).

### Myelin Loss in DAI

Gross morphologic evaluations were investigated to assess myelin loss using LFB staining. Myelin was apparent as a blue substrate reaction in coronal brain sections, particularly in the corona radiata and CC. In control rats, myelin was organized in a continuous and regular pattern. After impact injury, reduced LFB staining was observed in corona radiata and brain stem. Additionally, DAI rats had a disorganized myelin stained with LFB (Figure 1B–D).

LFB intensity might be affected following TBI, so we examined the MBP expression by western blot to further confirm the myelin loss in DAI. In the CC, there was a significant decrease in MBP expression at day 3, which recovered to control levels by day 7 post-DAI. Compared to the control rats, the MBP expression in brain stem was significantly decreased from day 1 post-injury in the brain stem, which further decreased at day 3 post-injury and remained decreased at day 7 (Figure 1E). These results are consistent with LFB staining, indicating loss of myelin following DAI.

### Increased Expression of Olig2-Positive Cells in DAI

**FIGURE 2:**
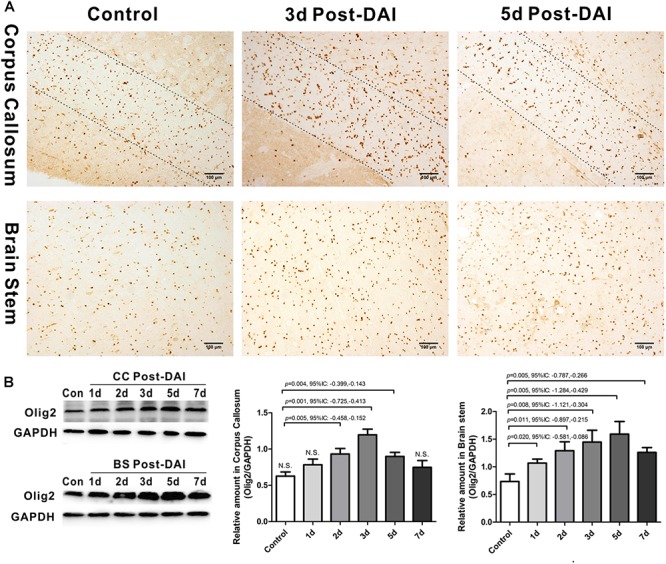
Increased expression of Olig2-positive cells in DAI. **(A)** Immunohistochemical results show that DAI induced the increased number of Olig2-positive cells in the CC and brain stem; **(B)** western blot analysis indicates that Olig2 expression was significantly increased throughout the first week after injury in the brain stem. The CC region exhibits significant Olig2 proliferation at 2, 3, or 5 days after injury. N.S. indicates no significant changes between DAI group and control group.

Olig2 was used here as a general marker of oligodendrocyte lineage cells because Olig2 is expressed in both oligodendrocyte precursor cells and mature oligodendrocytes. In control rats, a basal level of Olig2-positive cells was observed in both the CC and the brain stem. Olig2-positive cells showed a rounded morphology with staining in the nucleus. There was an increase in Olig2-positive cell numbers in both the CC and brain stem following DAI (Figure 2A). We further quantified expression of Olig2 by western blot analysis. Olig2 expression was significantly increased throughout the first week after injury in the brain stem. Meanwhile, in the CC increased Olig2 expression was only observed in injured rats at day 2, 3, and 5 versus the control rat (Figure 2B).

### Oligodendrocyte Apoptosis Following DAI

**FIGURE 3:**
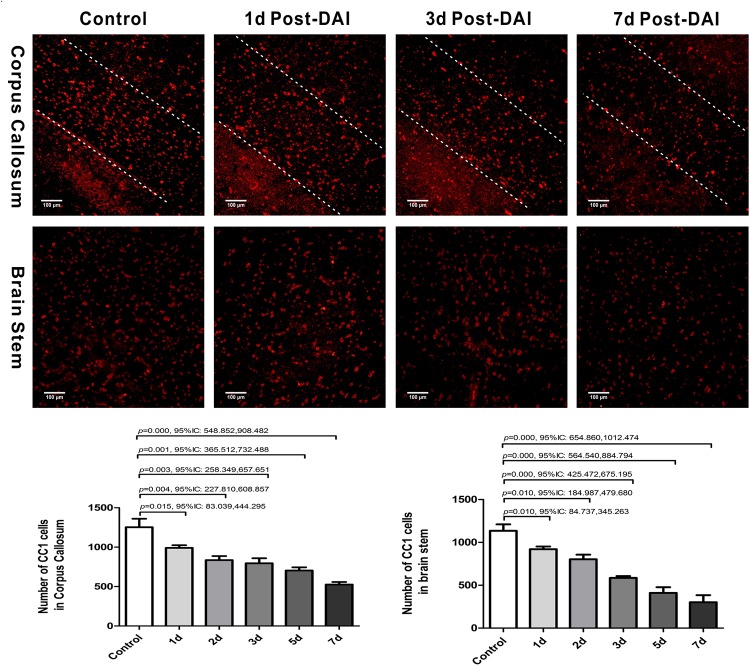
Diffuse axonal injury results in a loss of CC-1 immunoreactivity. Immunofluorescent results showed that numbers of CC-1+ immunostained cells within the injured regions of interest were significantly decreased at each of the time points after injury.

We then evaluated the number of oligodendrocyte by staining for the mature oligodendrocyte marker CC-1. CC-1 staining was localized to the cytoplasm and a large number of CC-1-positive cells were counted within the CC and brain stem in the control group. Numbers of CC-1 immunoreactive cells within the injured regions of interest were significantly decreased at each of the time points after injury (Figure 3).

**FIGURE 4:**
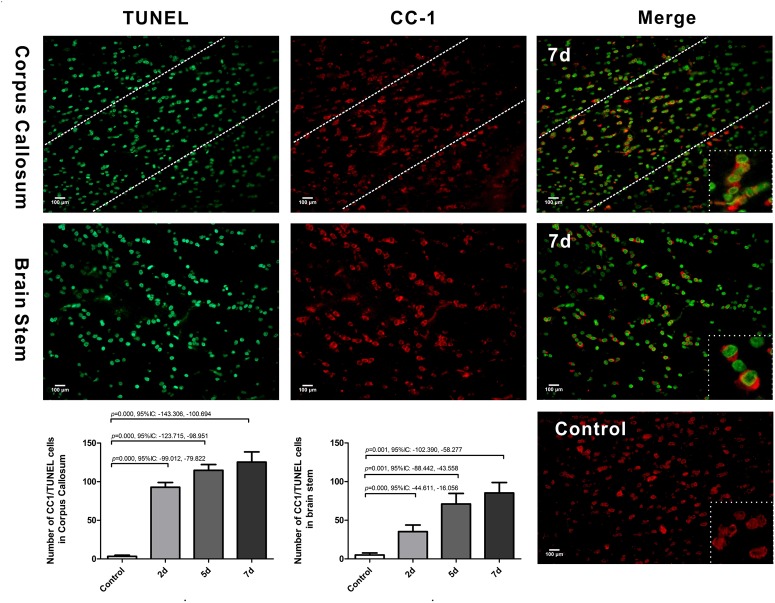
Oligodendrocyte apoptosis in DAI. Co-labeling of the mature oligodendrocyte marker CC-1 (red) and TUNEL (green). The control showed rare CC1/TUNEL co-labeled cells. Compared with the control, DAI significantly increased the number of CC1/TUNEL-positive cells.

We further examined oligodendrocyte apoptosis by using double immunofluorescence staining for CC-1 and TUNEL. The majority of TUNEL-labeled cells were seen within white matter tracts. In control rats, there were only rare CC-1/TUNEL co-labeled cells. Compared with the control, the number of CC-1/TUNEL-positive cells in the CC and brain stem were significantly increased at day 2 post-DAI, and it further increased with the continuous extension of injury time (Figure 4). The persistent increase in the number of CC-1/TUNEL co-labeled cells during myelin loss suggests that myelin loss is likely due to oligodendrocyte apoptosis in this moderate DAI model.

### Activation of Microglia/Macrophage Following DAI

**FIGURE 5:**
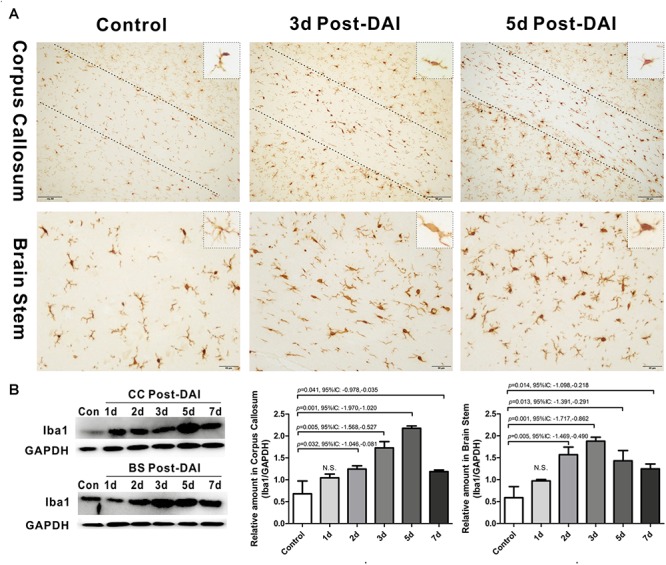
Activation of microglia/macrophages in DAI. **(A)** Immunohistochemical results show that DAI induced an increased number of Iba1-positive cells in the CC and brain stem; **(B)** western blot analysis indicates that Iba1 expression was significantly increased from 2 to 7 days after injury in both the CC and brain stem. N.S. indicates no significant changes between DAI group and control group.

Iba1 immunohistochemistry was used to characterize microglia/macrophage activation after DAI. Iba1 positive cells were rarely observed in the white matter tracts of the control rats. Compared to the control, Iba1 expression with markedly increased staining intensity was evident in the CC and brain stem in DAI rats. Moreover, different hypertrophic morphology of the Iba1-immunolabeled cells was identified after injury (Figure 5A). Similarly, the western blot results also showed increased expression of Iba1 from days 2 to 7 after injury in the CC and brain stem (Figure 5B).

### Mild Astrogliosis in the CC Following DAI

**FIGURE 6:**
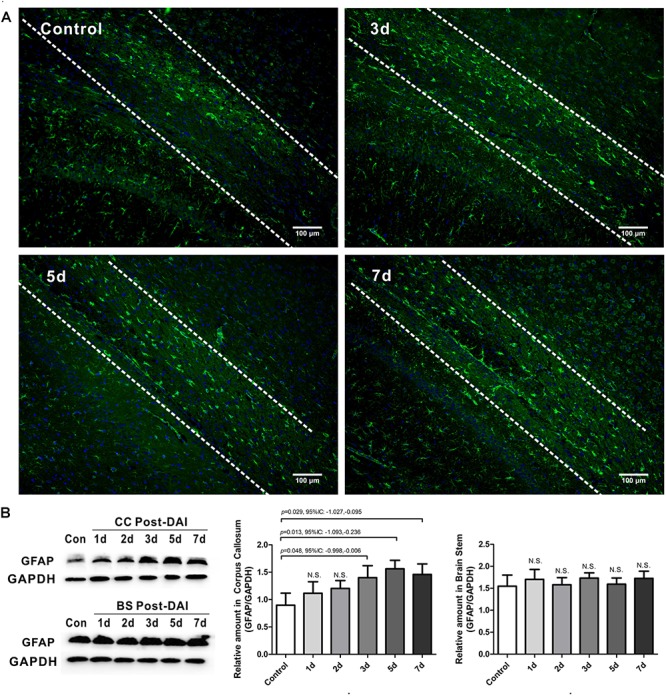
Mild astrogliosis in the CC. **(A)** Immunofluorescence for GFAP shows reactive astrocyte is significantly increased in CC; **(B)** western blot analysis indicates that GFAP expression is significantly increased in the CC at 3, 5, and 7 days post-DAI. However, there was no effect of DAI on GFAP expression in brain stem. N.S. indicates no significant changes between DAI group and control group.

GFAP was used to study the expression of astrocyte. GFAP showed widespread immunoreactivity in the cortex and white matter tracts of the control rats. Following DAI, GFAP immunoreactivity showed marked staining of dense astrocyte processes within the CC (Figure 6A). Quantification by western blot analysis confirmed that GFAP expression is significantly increased in the CC at 3, 5, and 7 days post-DAI. However, there was no effect of DAI on GFAP expression in the brain stem (Figure 6B).

### Ultrastructural Evidence of Axon Damage and Myelin Abnormalities After DAI

**FIGURE 7:**
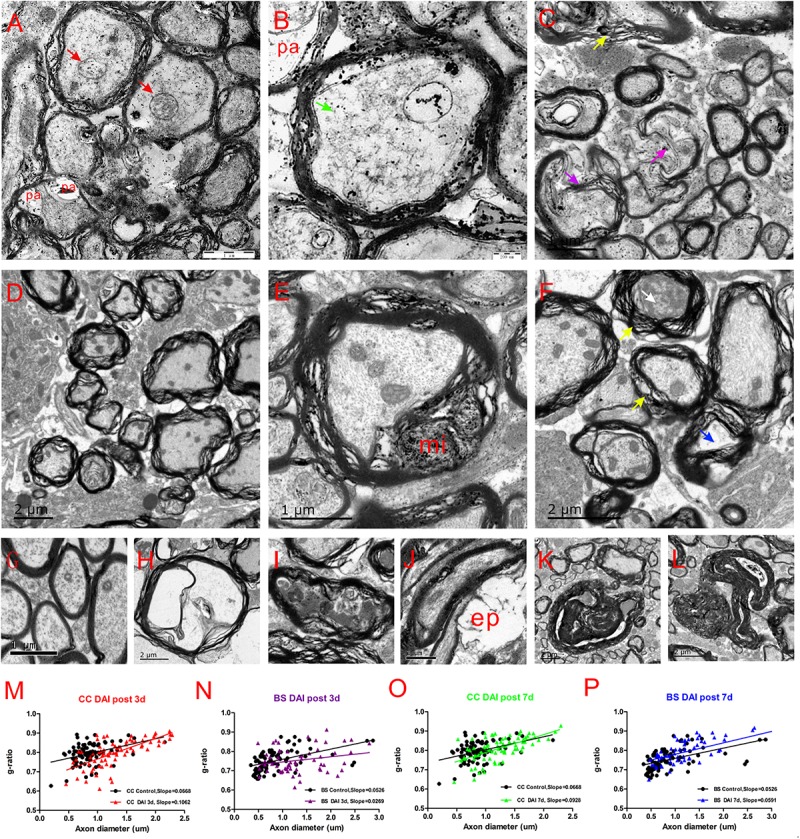
Ultrastructural changes in the CC and brain stem after DAI. **(G)** A normal distribution of myelinated fibers in the control; **(A,B)** at 3 days after DAI, the CC exhibits reduced cytoskeleton (green arrow), highly swollen mitochondria (red arrow), and periaxonal spaces (pa); **(D)** at 3 days after DAI, the brain stem exhibits marked myelin abnormalities along viable axons. The myelin extends outside (external protrusions, ep, **J**) or inside (myelin intrusions, mi, **E**); at 7 days after DAI, both the CC **(C)** and brain stem **(F)** regions exhibit obvious degenerating axons and abnormal myelin at 7 days after DAI. There is light degeneration (**H** and blue arrow) and dark degeneration (**I** and white arrow). Typical myelin abnormalities included diffuse separation of the sheath layers (yellow arrowheads), deterioration (purple arrow), collapse of the myelin **(K)** and excessive myelin figures **(L)**; **(M–P)** the change of g-ratio (axon/fiber diameter) in the CC and brain stem.

Electron microscopy of the CC and brain stem was used to investigate the ultrastructural changes of myelin and axon after DAI. In control rats, the myelin sheath was compact, with regularly organized myelin lamellae. The axolemma adhered tightly to the inner layer of myelin sheath and the axoplasmic contents such as microtubes, neurofilaments, and mitochondria were distributed regularly (Figure 7G).

At day 3 after injury, the CC regions exhibited a focal disorganization of the myelin sheath. It is noteworthy that most axons are irregular in profile and contains reduced cytoskeleton (Figure 7A,B). The patches of neurofilaments indicated partial proteolysis of the axonal cytoskeleton. Moreover, a number of periaxonal spaces (pa) occur between the axon and the myelin sheath. Highly swollen mitochondria within the axoplasm contains central lacuna. Compared with the control, the g ratio relative to the axon diameter in the DAI group is increased, which may be associated with a larger caliber of swollen axons (Figure 7M).

Unlike CC, the brain stem regions exhibit widespread delamination of myelin lamellae at day 3 following DAI (Figure 7D). The myelin extended either outside (external protrusions, ep, Figure 7J) or inside (myelin intrusions, mi, Figure 7E), while the axonal cytoskeleton was generally organized and mitochondria appeared intact. In the brain stem, an increased thickness of myelin caused by disrupted myelin lamellae led to a reduction in the value of the g ratio following DAI (Figure 7N).

At day 7 after injury, obvious degenerating axons and abnormal myelin were observed in both the CC (Figure 7C) and brain stem regions (Figure 7F). Degenerating axons exhibited cytoskeletal dissolution (light degeneration, Figure 7H) or amorphous electron dense material (dark degeneration, Figure 7I). Meanwhile, DAI rats also showed a marked feature of myelin pathology. Most of myelin lamellae were separated diffusely and disconnected locally. Myelin sheaths often collapsed around a degenerating axon or back onto themselves if the accompanying axon was disappeared (Figure 7K). Moreover, excessive myelin figures were also observed (Figure 7L). Both the CC (Figure 7O) and brain stem (Figure 7P) showed an increased slope in the g-ratio plots at 7 days after injury.

## Discussion

Formerly, DAI has been classically regarded as a primarily axonal degenerative disorder and the myelin sheath collapses as the axon degenerates. However, our present study demonstrates that damage can be initiated in myelin along intact axons in the brain stem following moderate DAI. Oligodendrocyte selective vulnerability and subsequent demyelination may contribute to axonal degeneration in the brain stem.

Currently, the severity of DAI is classified based on the areas of white matter with traumatic axonal injury (TAI) (Kim et al., 2008). Both DAI and TAI refer to studies of TBIs where axonal injury is the dominant component. They are often used interchangeably, although DAI occupies the more severe end of the spectrum of diffuse trauma-induced brain injury (Geddes et al., 2010). Mild DAI involves lesions mainly in the corona radiata. Moderate DAI includes corona radiata with the addition of CC. Severe DAI involves these sites as well as the brainstem tracts. In the present study, we used a modified Marmarou method to create a moderate DAI model. Depending on different direction and intensity of mechanical force transmitted to the different brain regions, it is expected that different injured regions may exhibit separated pathological processes involving axons and myelin. Thus, both the CC and brain stem were selected to be studied.

In the present study, our results clearly demonstrated that moderate DAI leads to myelin loss, revealed by LFB staining, MBP western blot analysis and electron microscopy. Moreover, DAI induces a persistent reduction in mature oligodendrocytes, with marked increases in the numbers of apoptotic oligodendrocytes for up to 7 days post-DAI. Thus, we suggest that the delayed oligodendrocyte death may be a significant factor underlying myelin loss in DAI. Myelin allows the rapid transmission of information that is needed for normal emotional, cognitive, and behavioral function (Deoni et al., 2011; Hong et al., 2015). Consequently, oligodendrocyte loss and myelin degeneration can impair saltatory conduction and modify circuit function, which is a potential factor underlying the slow information-processing speed in patients with DAI.

Oligodendrocyte progenitor cells (OPCs), as a source of new oligodendrocytes, can be stimulated to proliferate, migrate and differentiate in response to demyelination (Zhang et al., 2001; Jankovski et al., 2010). Our present results show that the number of Olig2-positive cells was increased in the first week post-injury. In a TAI model caused by central fluid percussion injury, Flygt et al. (2017) found that the numbers of both EdU/DAPI/Olig2- and EdU/DAPI/NG2-positive cells were increased and proposed that TAI induces a transient proliferative response of residing OPCs. Moreover, Xu et al. (2015) transplanted OPCs into the deep sensorimotor cortex of DAI rats and found that OPCs migrate en masse along white matter tracts and differentiate extensively into ensheathing oligodendrocytes. These data suggest that proliferating OPCs may replace dead oligodendrocytes and contribute to myelin remodeling and regeneration following TAI.

The capacity for spontaneous remyelination in injured tissues can be impaired by many factors, such as inflammatory cytokine released by activated microglia and glial scars formed by astrogliosis (Gallo and Armstrong, 2008; Clarner et al., 2012). Similar to our results, Jia et al. (2012) demonstrated that DAI rats also exhibit microglial activation in the CC during the acute stage, which plays an important role in secondary pathologic changes in DAI. In our study, we found that astrogliosis is relatively mild during this first week, which is similar to Sullivan’s findings in a mouse TAI model (Sullivan et al., 2013). The mild activated astrocytes can secrete a range of factors including neurotrophic factors, growth factors and cytokines that stimulate re/myelination by promoting OPC survival, proliferation and/or maturation (Kiray et al., 2016). Thus, mild astrogliosis in the context of myelin loss plays an important role in promoting the recovery of CNS function.

The ultrastructure analysis with electron microscopy provides further evidence of pathological changes of axons and myelin post-DAI. Notably, larger myelinated nerve fibers are the primary damaged objects in the early period following DAI as the g ratio rapidly changes in larger nerve fibers. Secondly, neither axon degeneration nor myelin loss occur within a narrow time frame, but in the secondary injury stage. Lastly, different injured regions exhibit separated pathological processes involving axons and myelin.

Our ultrastructural analysis demonstrated that CC was characterized by distinct axonal pathology at day 3 post-injury. Next to the impact site, the long axonal projections that traverse CC are simulated by forces of tension, torsion and compression, and consequently cause axonemal and cytoskeletal disruption as the cell injury triggers mechanically (Montanino and Kleiven, 2018). Following the initial damage, the complex secondary insults, including calcium overload, mitochondrial dysfunction, oxidative stress, glutamate excitotoxicity, eventually cause Wallerian degeneration and irreversible axonal disconnection (Chen et al., 2002). Notably, we also found that axonal injury also leads to structural damage to the adjacent myelin membrane at 7 days post-injury. It was hypothesized that calpain leak from damaged axons may mediate detachment of MBP from myelin membranes, leading to an instability of the myelin sheath and the initiation of demyelination (Liu et al., 2010).

Unlike the CC, the brain stem, which is farther from the impact site and bears less intensity and displacement of initial mechanical force, exhibited marked myelin abnormalities along viable axons at day 3 post-injury. We speculate that oligodendrocytes with the highest metabolic rate and limited antioxidants are more sensitive to secondary insults than axons. Interestingly, myelin-oligodendrocyte disruption further destroys axonal integrity at day 7 after injury in the brain stem. In fact, demyelination has severe consequences for the axonal partner. In addition to insulating the axons by myelination, myelin-oligodendrocytes deliver critical energy substrates to the axons through the monocarboxylate transporter 1 (MCT1) (Zhou et al., 2017). So, the most immediate effect of myelin-oligodendrocyte unit damage is the loss of trophic and metabolic support for the axon. Moreover, the loss of myelin also causes an increased energy demand of axons. Following demyelination, sodium channels redistribute across the entire axonal surface, and the axon would need more energy to maintain ion gradients (Waxman et al., 2004). Finally, myelin debris, served as neurotoxic mediators, expose the axons to inflammatory cytokines (Simons et al., 2014). Thus, myelin disruption caused by delayed oligodendrocyte death may also contribute to severe axonal degeneration.

The above ultrastructure observations revealed a complex interplay between axonal degeneration and myelin damage. So, we suggest that axon-myelin should be regarded as a highly integrated structural and functional unit, rather separate entities. In addition, depending on the separate pathological processes of DAI in CC (myelin sheath degeneration subsequent to axonal transection) vs. the brain stem (axonal degeneration subsequent to myelin disruption), DAI could be further divided into a different pathological pattern. As degenerative axons are difficult to repair while remyelination is feasible, promoting myelin regeneration may be an important target for therapeutic interventions of DAI.

## Conclusion

As a whole, the findings presented in this study highlight the need to evaluate pathological changes in both axon damage and myelin degeneration following DAI. A persistent apoptosis of mature oligodendrocytes within the CC and brain stem indicate oligodendrocyte-selective vulnerability in DAI. The axonal degeneration secondary to demyelination in the brain stem may be another pathological pattern of DAI. Defining the cause of ongoing oligodendrocyte death and promoting myelin regeneration may be important targets for therapeutic interventions of DAI.

## Author Contributions

JK and GZ designed the study. JM, ML, TW, XL, and MB performed the research, collected and analyzed the data. JM wrote the manuscript. All authors discussed the results and revised the manuscript.

## Conflict of Interest Statement

The authors declare that the research was conducted in the absence of any commercial or financial relationships that could be construed as a potential conflict of interest.
